# *N*-(4-Chloro­butano­yl)-*N*′-[2-(trifluoro­meth­yl)phen­yl]thio­urea

**DOI:** 10.1107/S1600536812008859

**Published:** 2012-03-10

**Authors:** Mohd Sukeri Mohd Yusof, Nur Farhana Embong, Suhana Arshad, Ibrahim Abdul Razak

**Affiliations:** a Department of Chemical Sciences, Faculty of Science and Technology, Universiti Malaysia Terengganu, Mengabang Telipot, 21030 Kuala Terengganu, Malaysia; b School of Physics, Universiti Sains Malaysia, 11800 USM, Penang, Malaysia

## Abstract

In the title compound, C_12_H_12_ClF_3_N_2_OS, the dihedral angle between the benzene ring and the thio­urea fragment is 69.41 (5)°. The thio­urea N—H atoms adopt an *anti* conformation, such that one of them forms an intra­molecular N—H⋯O hydrogen bond, generating an *S*(6) ring. In the crystal, both N—H groups form inversion dimers, one *via* a pair of N—H⋯S hydrogen bonds and one *via* a pair of N—H⋯O hydrogen bonds. These lead to *R*_2_^2^(8) and *R*_2_^2^(12) loops, respectively. Weak C—H⋯Cl, C—H⋯F, C—H⋯S and π–π [centroid–centroid separation = 3.7098 (6)Å and slippage = 1.853 Å] inter­actions also occur.

## Related literature

For a related structure and background to thio­urea derivatives, see: Yusof *et al.* (2011 ▶). For related structures, see: Khawar Rauf *et al.* (2006 ▶); Yusof *et al.* (2007 ▶). For hydrogen-bond motifs, see: Bernstein *et al.* (1995 ▶). For the stability of the temperature controller used in the data collection, see: Cosier & Glazer (1986 ▶).
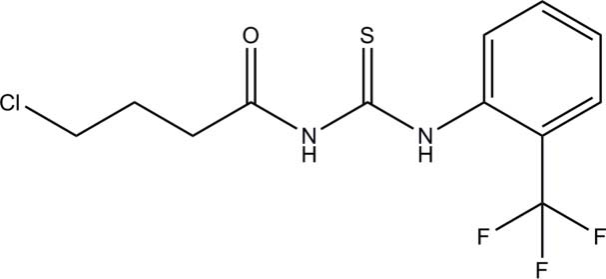


## Experimental

### 

#### Crystal data

C_12_H_12_ClF_3_N_2_OS
*M* *_r_* = 324.75
Triclinic, 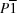

*a* = 7.8622 (1) Å
*b* = 8.9073 (1) Å
*c* = 11.0341 (1) Å
α = 113.687 (1)°
β = 103.419 (1)°
γ = 95.653 (1)°
*V* = 672.18 (2) Å^3^
*Z* = 2
Mo *K*α radiation
μ = 0.47 mm^−1^
*T* = 100 K
0.41 × 0.19 × 0.15 mm

#### Data collection

Bruker SMART APEXII CCD diffractometer
Absorption correction: multi-scan (*SADABS*; Bruker, 2009) ▶ *T*_min_ = 0.829, *T*_max_ = 0.932
18148 measured reflections
4884 independent reflections
4304 reflections with *I* > 2σ(*I*)
*R*_int_ = 0.021

#### Refinement

*R*[*F* ^2^ > 2σ(*F* ^2^)] = 0.030
*wR*(*F* ^2^) = 0.078
*S* = 0.97
4884 reflections
189 parameters
H atoms treated by a mixture of independent and constrained refinement
Δρ_max_ = 0.49 e Å^−3^
Δρ_min_ = −0.32 e Å^−3^

### 

Data collection: *APEX2* (Bruker, 2009) ▶; cell refinement: *SAINT* (Bruker, 2009) ▶; data reduction: *SAINT* ▶; program(s) used to solve structure: *SHELXTL* (Sheldrick, 2008 ▶); program(s) used to refine structure: *SHELXTL*; molecular graphics: *SHELXTL*; software used to prepare material for publication: *SHELXTL* and *PLATON* (Spek, 2009 ▶).

## Supplementary Material

Crystal structure: contains datablock(s) global, I. DOI: 10.1107/S1600536812008859/hb6655sup1.cif

Structure factors: contains datablock(s) I. DOI: 10.1107/S1600536812008859/hb6655Isup2.hkl

Supplementary material file. DOI: 10.1107/S1600536812008859/hb6655Isup3.cml

Additional supplementary materials:  crystallographic information; 3D view; checkCIF report

## Figures and Tables

**Table 1:** Hydrogen-bond geometry (Å, °)

*D*—H⋯*A*	*D*—H	H⋯*A*	*D*⋯*A*	*D*—H⋯*A*
N1—H1*N*1⋯O1	0.855 (16)	1.971 (17)	2.6486 (12)	135.4 (16)
N1—H1*N*1⋯O1^i^	0.855 (16)	2.514 (17)	3.2273 (12)	141.6 (14)
N2—H1*N*2⋯S1^ii^	0.849 (17)	2.682 (17)	3.5079 (10)	164.7 (14)
C2—H2*A*⋯Cl1^iii^	0.95	2.82	3.5535 (11)	135
C3—H3*A*⋯F1^iv^	0.95	2.47	3.2617 (13)	140
C9—H9*A*⋯S1^ii^	0.99	2.84	3.7829 (10)	159

Symmetry codes: (i) 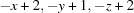; (ii) 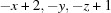; (iii) 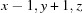; (iv) 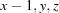.

**Fig. 1.**
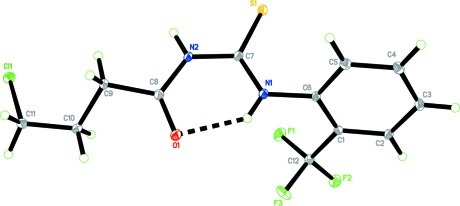
The molecular structure of the title compound with 30% probability displacement ellipsoids.

**Fig. 2.**
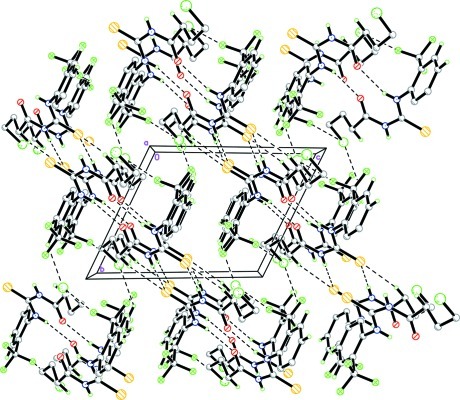
The crystal packing of the title compound. The H atoms not involved in the intermolecular interactions (dashed lines) have been omitted for clarity.

## supplementary crystallographic information

### Comment

As part of our ongoing studies of thiourea derivatives we now describe the title compound. It is analogous to the previously reported *N*-(4-chlorobutanoyl)-*N*'- (2-fluorophenyl)thiourea (Yusof *et al.*, 2011) except the fluoro atom is replaced by trifluoromethyl atom.

In the molecular structure (Fig. 1), the benzene ring (C1–C6) is essentially planar with maximum deviation of 0.011 (1) Å at atom C5. The intramolecular N1—H1N1···O1 hydrogen bond (Table 1) generates *S*(6) ring motifs (Berstein *et al.*, 1995). The bond lengths and angles are within normal ranges and are comparable to the related structures (Khawar Rauf *et al.*, 2006; Yusof *et al.*, 2007).

The crystal packing is shown in Fig. 2. *R*^1^_2_(6), *R*^2^_2_(8), *R*^2^_2_(12) ring motifs (Berstein *et al.* 1995) are formed by intermolecular N2—H1N2···S1, N1—H1N1···O1 and C9—H9A···S1 (Table 1) hydrogen bonds, respectively. Intermolecular C2—H2A···Cl1 and C3—H3A···F1 (Table 1) interactions linked the molecules into three-dimensional network. π–π interaction [*Cg*1···*Cg*1 (-1 - *x*, 1 - *y*, 1 - *z*) = 3.7098 (6) Å;] are also observed [*Cg*1: C1–C6].

### Experimental

An equimolar amount of 2-(trifluoromethyl)aniline (1.14 g, 7.09 mmol) in 20 ml acetone was added drop-wise into a stirring acetone solution (75 ml) containing 4-chlorobutanoylchloride (1.00 g, 7.09 mmol) and ammonium thiocyanate (0.54 g, 7.09 mmol). The mixture was refluxed for 1 h. Then, the solution was filtered-off and left to evaporate at room temperature to yield colourless needles.

### Refinement

N-bound H atoms was located from the difference map and refined freely, [N–H = 0.856 (17) and 0.849 (15) Å]. The remaining H atoms were positioned geometrically [C–H = 0.95 or 0.99 Å] and refined using a riding model with *U*_iso_(H) = 1.2 *U*_eq_(C).

### Crystal data

**Table:**

C_12_H_12_ClF_3_N_2_OS	*Z* = 2
*M**_r_* = 324.75	*F*(000) = 332
Triclinic, *P*1	*D*_x_ = 1.605 Mg m^−^^3^
Hall symbol: -P 1	Mo *K*α radiation, λ = 0.71073 Å
*a* = 7.8622 (1) Å	Cell parameters from 9937 reflections
*b* = 8.9073 (1) Å	θ = 2.5–32.6°
*c* = 11.0341 (1) Å	µ = 0.47 mm^−^^1^
α = 113.687 (1)°	*T* = 100 K
β = 103.419 (1)°	Needle, colourless
γ = 95.653 (1)°	0.41 × 0.19 × 0.15 mm
*V* = 672.18 (2) Å^3^	

### Data collection

**Table:**

Bruker SMART APEXII CCD diffractometer	4884 independent reflections
Radiation source: fine-focus sealed tube	4304 reflections with *I* > 2σ(*I*)
Graphite monochromator	*R*_int_ = 0.021
φ and ω scans	θ_max_ = 32.6°, θ_min_ = 2.1°
Absorption correction: multi-scan (*SADABS*; Bruker, 2009)	*h* = −11→11
*T*_min_ = 0.829, *T*_max_ = 0.932	*k* = −13→12
18148 measured reflections	*l* = −15→16

### Refinement

**Table:**

Refinement on *F*^2^	Primary atom site location: structure-invariant direct methods
Least-squares matrix: full	Secondary atom site location: difference Fourier map
*R*[*F*^2^ > 2σ(*F*^2^)] = 0.030	Hydrogen site location: inferred from neighbouring sites
*wR*(*F*^2^) = 0.078	H atoms treated by a mixture of independent and constrained refinement
*S* = 0.97	*w* = 1/[σ^2^(*F*_o_^2^) + (0.0365*P*)^2^ + 0.3295*P*] where *P* = (*F*_o_^2^ + 2*F*_c_^2^)/3
4884 reflections	(Δ/σ)_max_ < 0.001
189 parameters	Δρ_max_ = 0.49 e Å^−^^3^
0 restraints	Δρ_min_ = −0.32 e Å^−^^3^

### Special details

**Table:**

Experimental. The crystal was placed in the cold stream of an Oxford Cryosystems Cobra open-flow nitrogen cryostat (Cosier & Glazer, 1986) operating at 100.0 (1) K.
Geometry. All e.s.d.'s (except the e.s.d. in the dihedral angle between two l.s. planes) are estimated using the full covariance matrix. The cell e.s.d.'s are taken into account individually in the estimation of e.s.d.'s in distances, angles and torsion angles; correlations between e.s.d.'s in cell parameters are only used when they are defined by crystal symmetry. An approximate (isotropic) treatment of cell e.s.d.'s is used for estimating e.s.d.'s involving l.s. planes.
Refinement. Refinement of *F*^2^ against ALL reflections. The weighted *R*-factor *wR* and goodness of fit *S* are based on *F*^2^, conventional *R*-factors *R* are based on *F*, with *F* set to zero for negative *F*^2^. The threshold expression of *F*^2^ > σ(*F*^2^) is used only for calculating *R*-factors(gt) *etc*. and is not relevant to the choice of reflections for refinement. *R*-factors based on *F*^2^ are statistically about twice as large as those based on *F*, and *R*- factors based on ALL data will be even larger.

### Fractional atomic coordinates and isotropic or equivalent isotropic displacement parameters (Å^2^)

**Table:**

	*x*	*y*	*z*	*U*_iso_*/*U*_eq_	
Cl1	1.66943 (3)	0.08190 (3)	0.87826 (3)	0.01972 (6)	
S1	0.77778 (4)	0.12238 (3)	0.48626 (3)	0.01851 (6)	
F1	0.97525 (9)	0.59829 (9)	0.65758 (8)	0.02506 (15)	
F2	0.86578 (9)	0.81937 (8)	0.70633 (8)	0.02360 (14)	
F3	0.97036 (10)	0.74396 (10)	0.86623 (7)	0.02883 (16)	
O1	1.11812 (11)	0.39045 (10)	0.93786 (8)	0.02277 (17)	
N1	0.83210 (12)	0.36357 (10)	0.74177 (9)	0.01434 (15)	
N2	1.02233 (11)	0.17737 (10)	0.71774 (9)	0.01377 (15)	
C1	0.69392 (13)	0.58116 (12)	0.69974 (9)	0.01248 (16)	
C2	0.54109 (13)	0.64135 (12)	0.66718 (10)	0.01409 (16)	
H2A	0.5523	0.7491	0.6683	0.017*	
C3	0.37240 (13)	0.54429 (13)	0.63303 (10)	0.01548 (17)	
H3A	0.2685	0.5864	0.6123	0.019*	
C4	0.35583 (14)	0.38496 (13)	0.62916 (10)	0.01655 (18)	
H4A	0.2404	0.3175	0.6038	0.020*	
C5	0.50789 (14)	0.32467 (12)	0.66239 (10)	0.01537 (17)	
H5A	0.4962	0.2162	0.6598	0.018*	
C6	0.67708 (13)	0.42288 (12)	0.69935 (9)	0.01262 (16)	
C7	0.87834 (13)	0.22901 (12)	0.65719 (10)	0.01306 (16)	
C8	1.13277 (14)	0.25600 (12)	0.85287 (10)	0.01538 (17)	
C9	1.27517 (14)	0.16553 (12)	0.88836 (10)	0.01620 (17)	
H9A	1.2920	0.0827	0.8021	0.019*	
H9B	1.2370	0.1043	0.9385	0.019*	
C10	1.45172 (14)	0.29236 (12)	0.97903 (10)	0.01529 (17)	
H10A	1.4875	0.3522	0.9272	0.018*	
H10B	1.4310	0.3765	1.0628	0.018*	
C11	1.60482 (14)	0.21825 (13)	1.02413 (10)	0.01720 (18)	
H11A	1.5689	0.1538	1.0726	0.021*	
H11B	1.7087	0.3100	1.0902	0.021*	
C12	0.87515 (13)	0.68537 (12)	0.73243 (11)	0.01615 (17)	
H1N2	1.050 (2)	0.0918 (19)	0.6625 (16)	0.021 (4)*	
H1N1	0.895 (2)	0.413 (2)	0.8270 (17)	0.026 (4)*	

### Atomic displacement parameters (Å^2^)

**Table:**

	*U*^11^	*U*^22^	*U*^33^	*U*^12^	*U*^13^	*U*^23^
Cl1	0.01900 (12)	0.01807 (11)	0.01890 (11)	0.00744 (9)	0.00681 (9)	0.00360 (9)
S1	0.01973 (12)	0.01899 (12)	0.01193 (11)	0.00983 (9)	0.00168 (9)	0.00240 (9)
F1	0.0166 (3)	0.0231 (3)	0.0371 (4)	0.0074 (3)	0.0159 (3)	0.0098 (3)
F2	0.0204 (3)	0.0169 (3)	0.0348 (4)	0.0028 (2)	0.0088 (3)	0.0125 (3)
F3	0.0184 (3)	0.0350 (4)	0.0206 (3)	−0.0043 (3)	−0.0044 (3)	0.0078 (3)
O1	0.0251 (4)	0.0206 (4)	0.0152 (3)	0.0129 (3)	0.0010 (3)	0.0015 (3)
N1	0.0152 (4)	0.0144 (3)	0.0114 (3)	0.0069 (3)	0.0024 (3)	0.0038 (3)
N2	0.0141 (4)	0.0126 (3)	0.0131 (3)	0.0064 (3)	0.0030 (3)	0.0039 (3)
C1	0.0115 (4)	0.0135 (4)	0.0110 (4)	0.0036 (3)	0.0033 (3)	0.0038 (3)
C2	0.0140 (4)	0.0149 (4)	0.0128 (4)	0.0056 (3)	0.0042 (3)	0.0048 (3)
C3	0.0117 (4)	0.0216 (4)	0.0130 (4)	0.0064 (3)	0.0040 (3)	0.0066 (3)
C4	0.0130 (4)	0.0209 (4)	0.0150 (4)	0.0023 (3)	0.0051 (3)	0.0070 (3)
C5	0.0157 (4)	0.0157 (4)	0.0147 (4)	0.0034 (3)	0.0051 (3)	0.0064 (3)
C6	0.0126 (4)	0.0138 (4)	0.0105 (4)	0.0053 (3)	0.0033 (3)	0.0039 (3)
C7	0.0138 (4)	0.0128 (4)	0.0129 (4)	0.0048 (3)	0.0043 (3)	0.0053 (3)
C8	0.0155 (4)	0.0154 (4)	0.0138 (4)	0.0060 (3)	0.0030 (3)	0.0052 (3)
C9	0.0157 (4)	0.0146 (4)	0.0155 (4)	0.0065 (3)	0.0011 (3)	0.0049 (3)
C10	0.0171 (4)	0.0127 (4)	0.0138 (4)	0.0051 (3)	0.0037 (3)	0.0037 (3)
C11	0.0163 (4)	0.0174 (4)	0.0140 (4)	0.0061 (3)	0.0033 (3)	0.0031 (3)
C12	0.0127 (4)	0.0155 (4)	0.0175 (4)	0.0032 (3)	0.0037 (3)	0.0051 (3)

### Geometric parameters (Å, º)

**Table:**

Cl1—C11	1.8038 (10)	C2—H2A	0.9500
S1—C7	1.6748 (10)	C3—C4	1.3947 (14)
F1—C12	1.3455 (12)	C3—H3A	0.9500
F2—C12	1.3404 (12)	C4—C5	1.3904 (14)
F3—C12	1.3430 (12)	C4—H4A	0.9500
O1—C8	1.2255 (12)	C5—C6	1.3905 (14)
N1—C7	1.3358 (12)	C5—H5A	0.9500
N1—C6	1.4298 (12)	C8—C9	1.5112 (14)
N1—H1N1	0.856 (17)	C9—C10	1.5305 (14)
N2—C8	1.3813 (13)	C9—H9A	0.9900
N2—C7	1.3921 (12)	C9—H9B	0.9900
N2—H1N2	0.849 (15)	C10—C11	1.5082 (14)
C1—C2	1.3935 (13)	C10—H10A	0.9900
C1—C6	1.4011 (13)	C10—H10B	0.9900
C1—C12	1.5013 (14)	C11—H11A	0.9900
C2—C3	1.3888 (14)	C11—H11B	0.9900
			
C7—N1—C6	123.49 (8)	O1—C8—N2	122.61 (9)
C7—N1—H1N1	118.1 (11)	O1—C8—C9	122.16 (9)
C6—N1—H1N1	118.3 (11)	N2—C8—C9	115.22 (8)
C8—N2—C7	127.83 (8)	C8—C9—C10	109.74 (8)
C8—N2—H1N2	116.8 (10)	C8—C9—H9A	109.7
C7—N2—H1N2	115.1 (10)	C10—C9—H9A	109.7
C2—C1—C6	119.79 (9)	C8—C9—H9B	109.7
C2—C1—C12	119.61 (9)	C10—C9—H9B	109.7
C6—C1—C12	120.59 (8)	H9A—C9—H9B	108.2
C3—C2—C1	120.22 (9)	C11—C10—C9	115.10 (8)
C3—C2—H2A	119.9	C11—C10—H10A	108.5
C1—C2—H2A	119.9	C9—C10—H10A	108.5
C2—C3—C4	119.88 (9)	C11—C10—H10B	108.5
C2—C3—H3A	120.1	C9—C10—H10B	108.5
C4—C3—H3A	120.1	H10A—C10—H10B	107.5
C5—C4—C3	120.14 (9)	C10—C11—Cl1	111.48 (7)
C5—C4—H4A	119.9	C10—C11—H11A	109.3
C3—C4—H4A	119.9	Cl1—C11—H11A	109.3
C4—C5—C6	120.12 (9)	C10—C11—H11B	109.3
C4—C5—H5A	119.9	Cl1—C11—H11B	109.3
C6—C5—H5A	119.9	H11A—C11—H11B	108.0
C5—C6—C1	119.81 (9)	F2—C12—F3	106.59 (8)
C5—C6—N1	119.42 (8)	F2—C12—F1	106.08 (8)
C1—C6—N1	120.72 (9)	F3—C12—F1	106.43 (8)
N1—C7—N2	116.42 (8)	F2—C12—C1	112.63 (8)
N1—C7—S1	124.76 (7)	F3—C12—C1	112.29 (8)
N2—C7—S1	118.81 (7)	F1—C12—C1	112.35 (8)
			
C6—C1—C2—C3	−0.66 (14)	C8—N2—C7—N1	−5.61 (15)
C12—C1—C2—C3	178.23 (9)	C8—N2—C7—S1	173.63 (8)
C1—C2—C3—C4	−1.03 (14)	C7—N2—C8—O1	−2.41 (17)
C2—C3—C4—C5	1.43 (14)	C7—N2—C8—C9	178.37 (9)
C3—C4—C5—C6	−0.13 (15)	O1—C8—C9—C10	−40.56 (14)
C4—C5—C6—C1	−1.56 (14)	N2—C8—C9—C10	138.66 (9)
C4—C5—C6—N1	176.03 (9)	C8—C9—C10—C11	178.93 (8)
C2—C1—C6—C5	1.95 (14)	C9—C10—C11—Cl1	65.16 (10)
C12—C1—C6—C5	−176.93 (9)	C2—C1—C12—F2	−9.09 (13)
C2—C1—C6—N1	−175.61 (8)	C6—C1—C12—F2	169.80 (8)
C12—C1—C6—N1	5.52 (13)	C2—C1—C12—F3	111.26 (10)
C7—N1—C6—C5	66.17 (13)	C6—C1—C12—F3	−69.86 (12)
C7—N1—C6—C1	−116.27 (11)	C2—C1—C12—F1	−128.80 (9)
C6—N1—C7—N2	−173.57 (9)	C6—C1—C12—F1	50.08 (12)
C6—N1—C7—S1	7.24 (14)		

### Hydrogen-bond geometry (Å, º)

**Table:**

*D*—H···*A*	*D*—H	H···*A*	*D*···*A*	*D*—H···*A*
N1—H1*N*1···O1	0.855 (16)	1.971 (17)	2.6486 (12)	135.4 (16)
N1—H1*N*1···O1^i^	0.855 (16)	2.514 (17)	3.2273 (12)	141.6 (14)
N2—H1*N*2···S1^ii^	0.849 (17)	2.682 (17)	3.5079 (10)	164.7 (14)
C2—H2*A*···Cl1^iii^	0.95	2.82	3.5535 (11)	135
C3—H3*A*···F1^iv^	0.95	2.47	3.2617 (13)	140
C9—H9*A*···S1^ii^	0.99	2.84	3.7829 (10)	159

Symmetry codes: (i) −*x*+2, −*y*+1, −*z*+2; (ii) −*x*+2, −*y*, −*z*+1; (iii) *x*−1, *y*+1, *z*; (iv) *x*−1, *y*, *z*.
